# Supplementary material for: Genome-wide association and multi-omics functional screens reveal the genetic architecture of foveal development
Source: medRxiv. 2026 Jun 12:2026.06.11.26355452. Preprint. [Version 1] doi: 10.64898/2026.06.11.26355452 (PMC13278201; doi:10.64898/2026.06.11.26355452)
Supplement: Supplementary file 1 [file NIHPP2026.06.11.26355452V1-supplement-1.pdf]

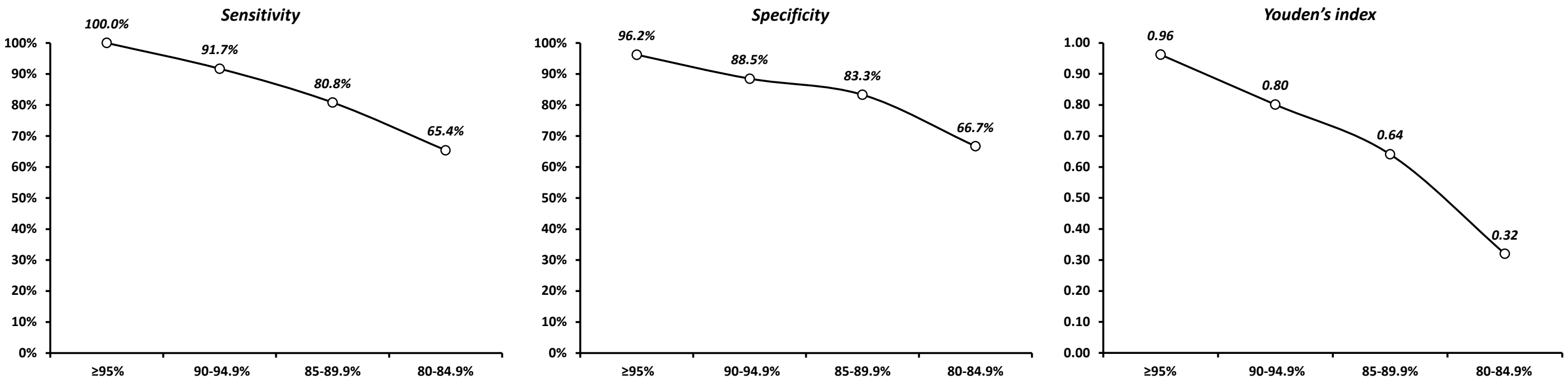

Supplementary Figure 1: Diagnostic performance by confidence threshold. Line graphs illustrating the Sensitivity, Specificity, and Youden's index of the model's classifications across four confidence threshold intervals (≥ 95%, 90-94.9%, 85-89.9%, and 80-84.9%). The ≥ 95% threshold was selected for final analysis as it yielded the optimal diagnostic performance (Sensitivity: 100.0%, Specificity: 96.2%, Youden's index: 0.96).
